# Increased Risk for Substance Use-Related Problems in Mild Intellectual Disability: A Population-Based Cohort Study

**DOI:** 10.1192/j.eurpsy.2022.243

**Published:** 2022-09-01

**Authors:** A. Påhlsson, S. Liu, M. Tideman, H. Larsson, P. Lichtenstein, A. Butwicka

**Affiliations:** 1 Karolinska Institutet, Medical Epidemiology And Biostatistics, Stockholm, Sweden; 2 Höhskolan i Halmstad, Akademin För Hälsa Och Välfärd, Halmstad, Sweden; 3 Örebro Universitete, Institutionen För Medicinska Vetenskaper, Örebro, Sweden

**Keywords:** Substance Use-Related Problems, intellectual disability, Population-Based Cohort Study

## Abstract

**Introduction:**

Intellectual disability (ID) has been linked to substance use-related problems (SUP). However, previous research is limited by the small sample sizes, lack of general population comparison and have not accounted for familial confoundings. The role of other psychiatric comorbidities also remains unknown.

**Objectives:**

To examine the risk of SUP in individuals with mild-ID and assess whether the associations depend on other psychiatric comorbidities, controlling for potential familial confounding.

**Methods:**

Population-based cohort study of individuals born in Sweden 1973-2003. We identified 19,078 individuals with mild-ID, 953,900 reference individuals from the general population, and 20,722 full-siblings of individuals with mild-ID. Conditional logistic regression models were used to compare individuals with mild-ID to the general population and their full-siblings regarding the risk of SUP, including alcohol and substance use disorders, alcohol and substance-related somatic diseases, substance-related crime, and substance-related death. Analyses were repeated stratified by the presence of psychiatric comorbidities.

**Results:**

Individuals with mild-ID had increased risks of any SUP (adjusted OR [95%CI]: 1.41 [1.35, 1.47]), compared to the general population, including alcohol-related somatic diseases (3.27 [1.92, 5.59]), alcohol (2.05 [1.91, 2.22]) and drug-use disorder (1.79 [1.69, 1.91]), and alcohol (1.36 [1.19, 1.49]) and drug-related crime (1.27 [1.19, 1.36]). The risk of SUP for individuals with mild ID was particularly elevated with comorbid mood (3.74 [3.47, 4.04]), anxiety (3.30 [3.09, 3.53]) and attention-deficit/hyperactivity disorders (2.61 [2.44, 2.80]). Increased risk of SUP remained significant when controlling for familial confounding.

**Conclusions:**

Individuals with mild-ID, especially those with other psychiatric comorbidities, are at increased risks of SUP.

**Disclosure:**

No significant relationships.

